# Iranian medical students’ tendency to migrate and its associated factors

**DOI:** 10.1186/s12909-023-04147-x

**Published:** 2023-04-12

**Authors:** Mohammad Taherahmadi, Mahboobeh Khabaz Mafinejad, Azadeh Sayarifard, Ali Akbari Sari, Parisa Farahani

**Affiliations:** 1 School of Medicine, Tehran University of Medical Sciences, Tehran, Iran; 2 Health Professions Education Research Center, Department of Medical Education, Education Development Center, Tehran University of Medical Sciences, Tehran, Iran; 3 Center for Academic and Health Policy, Tehran University of Medical Sciences, Tehran, Iran; 4 Department of Health Management and Economics, School of Public Health, Tehran University of Medical Sciences, Tehran, Iran

**Keywords:** Migration, Medical students, Iran

## Abstract

**Background:**

Medical staff migration is one of the challenges for both developed and developing countries affecting society’s health and welfare, which limits access to equity. Therefore, this study was designed and conducted to investigate the tendency to migrate and the factors affecting it among medical students of the Tehran University of medical sciences, Tehran, Iran, in 2019.

**Methods:**

This cross-sectional study was performed among 472 medical students using a valid questionnaire which was designed after reviewing the literature and using the opinions of experts. The tendency to migrate and its associated factors were analyzed and reported using the Pearson correlation test, independent t-test, one-way ANOVA test, Tukey post-hock test, and Kruskal–Wallis non-parametric test.

**Results:**

According to this study, the tendency to migrate was 6.13 ± 2.82 out of 10. While there was no significant relationship between age, marital status, medical educational phase and the tendency to migrate (*p* > 0.05); There was a significant relationship between willingness to migrate with variables of gender (*p* = 0.027), pre-university study region (*p* < 0.001), father’s academic degree (*p* = 0.007), mother’s academic degree (*p* < 0.001), having the relative abroad (*p* < 0.001), foreign trip experience (*p* < 0.001), foreign language skills (*p* < 0.001), number of published articles (*p* = 0.005) and Iran’s National Elite Foundation membership (*p* = 0.039).

**Conclusions:**

Females, elites, and those with higher socioeconomic state, previous exposure to foreign countries, the ability to speak foreign languages, and research activity are more likely to migrate. Considering the high tendency to migrate among Iranian medical students, urgent and severe strategies must be undertaken to solve this social and health problem.

## Background

Brain drain or elite migration is considered one of the most important challenges in the world. This phenomenon is defined as the transfer of human resources from a country where the country of origin needs the knowledge and expertise of its elites, but they prefer permanent or semi-permanent migration to other countries [1].

Currently, one of the most important factors of production is skilled and efficient human resources; thus, the elite migration plays an influential role in the development of countries [2, 3]. However in the health systems of some developing countries, the importance of these human resources has been neglected [4]. According to the literature, 25% of the medical and nursing staff of Canada, the United Kingdom, the United States of America, and Australia are educated abroad [5], which can threaten the development of the origin countries.

This phenomenon can cause many complications and problems for the country of origin. In a study that examines the costs of physicians’ migration, the cost of educating a general practitioner is estimated as 57,000 dollars, which in the case of migrating and working in another country for 30 years, costs about 450,000 dollars for the country of the origin [6]. Medical staff loss, reduced number of medical professors, increase in health care cost, increase in health services waiting time, lack of access to health facilities, decrease in health services quality, and as a result dissatisfaction are the other complications of medical staff migration [7–10].

Unfortunately, there are no comprehensive and documented data regarding the Iranian workforce migration, including the healthcare sector. World Health Organization report on the employment of physicians in 2017 showed that Iran, ranks seventh in terms of the number of migrant physicians; approximately 13,000 physicians have migrated to 35 OECD (Organization for Economic Co-operation and Development) countries, which represents an increase of about 40% since 2000 [11]. There are just some limited studies about Iranian medical staff’s intention to migrate, and to the best of our knowledge, there is no data available regarding it among medical students. Having accurate and systematic information enables us to recognize and analyze the Iranian medical students’ migration patterns, which will help us develop more effective policy decisions and solutions. Tehran University of Medical Sciences (TUMS) is considered the pioneer medical university in Iran [12]. In this research, we aimed to study the TUMS medical students’ tendency to migrate and its associated factors.

## Methods

### Participants

This cross-sectional, observational, questionnaire-based study was conducted among 472 medical Students of TUMS in Tehran, Iran, from September to November 2019. The inclusion criteria were to be Iranian and medical student of TUMS. Exclusion criteria were refusal to participate in the study, and incomplete questionnaire.

According to previous studies [13], the intention to migrate was 54.77%. With an acceptable standard deviation (SD) of 5% and an alpha error of 5%, the minimum required sample size of 380 participants was predicted. Since it was probable that some questionnaires remained unanswered or distorted, 480 questionnaires distributed. The undergraduate medical education curriculum in Iran consists of a seven-year degree program divided into four phases, including basic sciences, physiopathology, clerkship, and internship. Since the population was divided into four strata, the sample recruited from each stratum according to the population ratio of students. By the stratified clustered random method, the minimum number of samples required for each stratum was calculated. Then, some obligatory lecture classes among each cluster were selected randomly, and self-administered paper-based questionnaires were distributed among all participants immediately after the lectures (before they left the class). To maintain the privacy of students; the questionnaire was requested to be folded after completion. Because the data collection was done in person by distributing questionnaires right after the lectures, a high response rate with a high level of representativeness was achieved.

The study was submitted to and approved by the Tehran University of medical sciences ethics committee by the code of medical ethics IR.TUMS.MEDICINE.REC.1397.812. Participants were completely free to take part in the study, and verbal consent was obtained before questionnaires were distributed. Due to the type of study and anonymity of questionnaires, only verbal consent was obtained. All information was kept confidential by the researchers.

### Study tool

Based on the results of the literature review, the 74 basic items of the questionnaire were extracted. Twelve experts (clinicians and medical educationalists) invited to examine the content validity of items. They were requested to score each item from one to three with a three-point scale of “not necessary, useful but not essential, essential”‏. When the total number of experts was 12, the minimum content validity ratio (CVR) for each item was 0.56 [14]. Forty items with CVR lower than 0.56 were eliminated. Experts were also asked to examine each question on a four-point scale to determine the relevance of the items. Eventually; content validity index (CVI) computed and items with the CVI less than 0.79 were eliminated, which contains none of the remaining items of the CVR validation.

Afterward, we pilot tested the 34 remaining items with eight medical students to examine the face validity of items. According to their opinions, necessary changes were applied for better comprehensibility. The Cronbach’s Alpha was determined to be 0.87, which indicates the acceptable internal consistency of the items.

The questionnaire developed in three parts. Demographic and general information was collected in the first part (i.e., age, gender, marital status, pre-university study region, medical educational phase, parents’ academic degree, having a relative abroad, foreign trip experience, foreign language skills, number of published articles, and Iran’s National Elite Foundation (INEF) membership). In the next part, students were asked to rate their tendency to migrate from one (the lowest) to ten (the highest). The last part included additional questions about the push and pull factors affecting students’ tendency to migrate (findings are not reported here.). The questionnaire was in the Persian language, as Persian is the native language of Iranian medical students. All questionnaires were anonymous.

### Analysis

The data of this study were analyzed by version 22 of SPSS software. The continuous data were reported by mean and SD, and the categorical variables by numbers and percentages. Statistical tests used in this study included Pearson correlation test, independent t-test, one-way ANOVA test, Tukey post-hock test, and Kruskal–Wallis non-parametric test. Normality was checked for all the variables using statistical testing. According to Kolmogorov–Smirnov test, the variable of "number of published articles" appeared not to be normal, so we used non-parametric tests to examine its association with the tendency to migrate. Analyzes with a *P* value ≤ 0.05 were considered statistically significant in this study.

## Results

After discarding the distorted and incomplete questionnaires, a total of 472 questionnaires were collected and analyzed. Approximately half of the participants were male (55%), and most of them were single (91.49%). The majority of the students were in the age range of 19 to 27 years, with a mean age of 23.18 ± 6.13. Out of 472 participants, 148 individuals were in the basic sciences (31.4%), 80 in the physiopathology (16.9%), 123 in the clerkship (26.1%) and 121 in the internship (25.6%). The average tendency of students to migrate was 6.13 ± 2.82 out of 10. Other findings are mentioned in Table 1.

**Table 1:** Demographic characteristics and the tendency to migrate

Demographic variables	number (percentage)	Tendency to migrate ± SD	*P* value
Gender			
Female	210 (44.8%)	6.45 ± 2.66	0.027^a^
Male	259 (55.2%)	5.88 ± 2.92	
Marital status			
Single	430 (91.5%)	6.16 ± 2.80	0.319^a^
Married	40 (8.5%)	5.69 ± 3.12	
Educational phase			
Basic sciences	148 (31.4%)	6.32 ± 2.75	0.694^b^
Physiopathology	80 (16.9%)	5.87 ± 2.89	
clerkship	123 (26.1%)	6.15 ± 2.79	
Internship	121 (25.6%)	6.05 ± 2.92	
Pre-university study region			
Region one	184 (39.7%)	6.76 ± 2.77	< 0.001^b^
Region two	223 (48.0%)	5.84 ± 2.71	
Region three	57 (12.3%)	5.26 ± 3.00	
Father’s academic degree			
doctorate and more	155 (33%)	6.69 ± 2.70	0.007^b^
Master	102 (21.7%)	6.31 ± 2.78	
Bachelor	109 (23.2%)	5.74 ± 2.77	
undergraduate	103 (22.1%)	5.60 ± 2.96	
Mother’s academic degree			
doctorate and more	73 (15.5%)	7.36 ± 2.43	< 0.001^b^
Master	78 (16.6%)	6.07 ± 2.47	
Bachelor	170 (36.1%)	6.20 ± 2.92	
undergraduate	150 (31.8%)	5.48 ± 2.89	
Have first or second-degree relative living abroad			
None	230 (48.9%)	5.53 ± 2.88	< 0.001^b^
One relative	87 (18.5%)	6.35 ± 2.56	
More than one relative	153 (32.6%)	6.92 ± 2.69	
Foreign trip experience			
None	162 (34.4%)	5.38 ± 2.84	< 0.001^b^
One or two	144 (30.6%)	6.15 ± 2.72	
More than two	165 (35%)	6.83 ± 2.73	
Skills in English			
No	7 (1.5%)	5.42 ± 4.39	0.507^a^
Yes	465 (98.5%)	6.14 ± 2.80	
Skills in French			
No	445 (94.3%)	6.05 ± 2.84	0.013^a^
Yes	27 (5.7%)	7.44 ± 2.20	
Skills in Germany			
No	437 (92.6%)	5.96 ± 2.81	< 0.001^a^
Yes	35 (7.4%)	8.25 ± 2.07	
Skills in at least two foreign languages			
No	369 (78.3%)	5.87 ± 2.82	< 0.001^a^
Yes	102 (21.7%)	7.05 ± 2.65	
Published articles			
None	401 (85.3%)	5.96 ± 2.81	0.005^c^
One or two	52 (11.1%)	7.00 ± 2.63	
More than one	17 (3.6%)	7.41 ± 3.20	
Iran’s National Elite Foundation membership			
No	287 (61.1%)	5.91 ± 2.89	0.039^a^
Yes	183 (38.9%)	6.46 ± 2.69	

Frequency totals may be less than the stated number due to missing values

^a^Independent t test; ^b^ANOVA; ^c^Kruskal–Wallis

There was no significant relationship between age (Pearson’s correlation test), marital status, medical educational phase and the tendency to migrate (*p* > 0.05). Women appeared more likely tend to migrate (*p* = 0.027).

Based on the quality and availability of educational facilities in high schools, cities in Iran are classified into three pre-university study regions: one, two, or three. Accordingly; region one is considered the most privileged and region three the least. In this study, there was a significant association between the tendency to migrate and pre-university study region (*p* < 0.001). In order to determine the type of relationship, the Tukey post-hock test was used. Medical students from Region one tend to migrate more than students from region two and three (*p* = 0.003 and *p* = 0.001, respectively), but there was no significant difference between students from Region two and three in terms of their willingness to migrate (*p* = 0.338).

Father’s and mother’s academic degrees have a significant association with the tendency to migrate (*p* = 0.007 and *p* < 0.001, respectively). Participants who have a parent (father or mother) with a doctorate and more degree were more inclined to migrate, but there was no significant difference among other degrees according to the Tukey post-hock test (*p* > 0.05).

The study examined two variables related to respondents’ exposure to foreign countries: a relative living abroad and foreign trip experience, which both showed a significant association with the tendency to migrate (*p* < 0.001). Comparatively, students who had taken at least one foreign trip had a greater tendency to migrate than those who didn’t (*p* = 0.042). Students who had at least one first or second-degree relative living abroad, appeared to be more inclined to migrate compared with students who did not have any (*p* = 0.048). According to the Tukey post-hock test, there was no significant relationship between the tendency to migrate and the number of trips (less than three trips or three trips and more) (*p* = 0.079) and the number of relatives living abroad (one relative or more than one) (*p* = 0.284).

While there was no significant relationship between skills in English and the tendency to migrate (*p* = 0.507), those who declared their minimal skills in French (*p* = 0.013) or Germany (*p* < 0.001) were more inclined to migrate. On the other hand, those who had at least two foreign language skills ​​were more likely to migrate than those who had less (*p* < 0.001).

Having a published article (*p* = 0.005) and INEF membership (*p* = 0.039) appeared to be associated with the tendency to migrate. Different criteria considered for the membership of the individuals in INEF, one of the main criteria of which is the performance of individuals in the University entrance exam and Olympiad exams. Therefore, the majority of people who are members of this foundation can be considered as scientific pioneers of the country.

## Discussion

In light of the absence of documented data on medical students’ migration in Iran, we attempted to determine the factors involved.

This study questioned the tendency to migrate in the form of a range from one to ten, unlike many other studies that questioned the tendency to migrate as yes or no (willingness and unwillingness). As reported, the tendency of medical students to migrate is higher than the average, which is consistent with the results of the other studies on elite migration in Iran and other countries. According to Asadi et al., migration intention among Iranian medical staff is 54.77% [13]. Alaeddini et al. reported the Iranian physicians’ inclination to migrate at 53.3% [15]. Pakistanis medical students’ intention to migrate was reported to be 54% to 95% in different studies [16–18]. Since; Iran is projected to have a shortage of physicians by 2030, this high tendency to migrate is cause of concern [19].

Although there was no significant relationship between age and the tendency to migrate in this study, other studies have found that young people have a greater tendency to migrate [13, 20, 21]. Asadi et al. examined the tendency to migrate among medical students and graduates. They found that individuals under 35 years old showed significantly greater willingness to migrate than those over 35 years old [13]. In the present study, maybe due to the proximity of students’ age during their study period and the continuity of the undergraduate medical curriculum in Iran, no significant difference in the tendency to migrate regarding age was observed.

There are conflicting reports about the association between the tendency to migrate and the year of study. A study in Ireland found that those who plan to migrate at the senior stages of training (38%) were more than those at the junior stages (28%) [22]. Interns and students in higher years of the Addis Ababa University of Ethiopia have a higher tendency to migrate compared to those in lower years [23]. However in another study in Ghana, medical students’ intentions to migrate declined with increasing levels of training [24]. In this study, no significant relationship was found between students’ tendency to migrate and their medical educational phase. This may indicate that there are more factors affecting the intention to migrate than the year of study.

According to this study, women were more likely to migrate, which is inconsistent with other studies conducted in Iran. While Asadi et al. could not find any association between gender and intention to migrate [13], Alaaldini et al. demonstrated that men are more inclined to migrate [15]. Gender discrimination in Iran [25] and the presence of women’s rights laws in the destination countries may influence the desire of Iranian women to migrate. On the other hand, the existence of barriers to male migration, such as mandatory military service, adversely affects men’s decision to migrate [25]. Another consideration is the increasing age of the marriage and the age of the first childbirth in Iran in the last decade, which can ease the academic advancement for women and facilitate their migration plan [26–28]. The results concerning migration tendency and gender association are incompatible in different countries. In studies conducted in Ghana, Lebanon, Syria, Ethiopia, and Lithuania, men were more inclined to migrate [23, 24, 29–31]. In Poland this tendency was higher among women [20], and in Ireland there was no difference [22]. The difference in study design can be mentioned as the related causes, as well as differences in the political, religious, and socioeconomic status of different countries.

There was no significant relationship between the tendency to migrate and marital status in the present study; however, being single was a predictor of willingness to migrate in previous studies [13, 29, 32]. This can be due to the low proportion of married people in the study (8.5% vs. 91.5%). Also, some marriages at TUMS occur between students from the same field, which leads to similar attitudes and concerns between married and single individuals. It may cause no difference in terms of their desire to migrate and affects our study results accordingly.

The socioeconomic status of students and their families is another factor influencing their intention to migrate. Considering the uncertainty of Iran’s economic condition and the sensitivities toward the direct question of the financial status of individuals, three variables were used in this study to estimate the students’ socioeconomic status: Pre-university study region, Father’s academic degree, and Mother’s academic degree. Based on these, people who have higher socioeconomic status were inclined to migrate more. Different aspects of socioeconomic status should be considered in order to interpret this relationship. Migration as a time-consuming and costly process is usually possible with the financial support of family; thus, Expenses are considered as the barrier to migrate [33]. On the other hand, those with low incomes are more likely to enter the labor market earlier, which could pose a serious barrier to migration. Additionally, socioeconomic status potentially influences students’ tendency to migrate by affecting various factors, including having a relative abroad, foreign trip experience, and foreign language skills.

Since the migration decision is one of the most challenging and stressful decisions in any person’s life, prior exposure to foreign countries could affect the tendency to migrate through financial and advisory supporting roles. In our study, Students with at least one relative abroad or one foreign trip experience were more inclined to migrate. Various other studies mentioned that inclination to migrate is significantly influenced by the experience of being abroad [30, 34] and having friends or relatives living abroad [13, 30, 34, 35]. It should be noted that the above factors can affect the desire to migrate with indirect impacts on the socioeconomic status of individuals, which was mentioned earlier.

The ability to speak foreign languages also makes it possible for migrants to communicate more easily, which reduces their stress after the migration and also makes it easier for them to find better employment and educational opportunities. As a result, many studies have cited foreign language skills as a factor influencing the tendency to migrate [13, 34]. The results of this study indicate a significant relationship between the tendency to migrate and minimal skills in both French and German, but not in English. Since English is a part of the pre-university curriculum in Iran, the majority of students have a minimal understanding of it. On the other hand, other languages (e.g., German and French) are typically taught individually and privately, and several people learn them with the intent of migrating. As a result, the ability to understand another language could be assumed as an encouragement for migration or a consequence of planning to migrate. So the causality cannot be explained and each one can be a factor for the occurrence of the other.

As we discuss physicians’ migration, we must never forget that it is often about achieving higher scientific levels. Accordingly, many students who plan to study abroad try to improve their resumes. Research activity, which manifests itself in publication history, is one of the most significant factors affecting the resumes of individuals. According to the results of this study, people who had at least one published paper, compared to people without publications, are more inclined to migrate. It is in line with a study in Croatia, which demonstrated that students who were interested in research have more willing to migrate [21].

Even among the medical students of TUMS, which considered the pioneer medical university in Iran, members of INEF, who are regarded as the future scientific leaders of the country, are more inclined to migrate. In this sense, the foundation may have failed to support the elites properly and provide the necessary and sufficient conditions to pursue their scientific advancement. If the right policies are not adopted, there will be scientifically irreparable losses in the future, which should be a wake-up call for policymakers.

In terms of limitation, our study was conducted at the first-ranked university of medical sciences, which may exaggerate the actual tendency to migrate estimation among all medical students of Iran. Due to the cross-sectional nature of the study, it is possible that the tendency to migrate was influenced by the economic and political conditions at the time of data collection. Large sample size, including all medical educational phases, and assessing the majority of demographic variables are the strengths of this study.

It is recommended that similar studies be conducted at various intervals in other medical universities in Iran to obtain a more comprehensive view of the tendency to migrate among Iranian medical students.

## Conclusions

Iranian medical students’ tendency to migrate is more than average. Females, elites, and those with higher socioeconomic state, previous exposure to foreign countries, the ability to speak foreign languages, and research activity are more likely to migrate.

Due to the high tendency of migration among medical students and its undeniable threat to the health care system and the scientific future of the country, policymakers should seek severe and urgent strategies to solve this phenomenon. It is hoped that this study has been able to take a step towards solving this social and health problem by sensitizing decision-makers on the issue of migration by presenting factors affecting students’ willingness to migrate.

## Data Availability

The datasets generated and analyzed during the current study are available from the corresponding author on reasonable request.

## Abbreviations

OECD: Organization for Economic Co-operation and Development
TUMS: Tehran University of Medical Sciences
INEF: Iran’s National Elite Foundation
CVR: Content validity ratio
CVI: Content validity index
SD: Standard Deviation

## References

[1] Carrington WJ, Detragiache E. How extensive is the brain drain?. Finance and Development. 1999;36:46–9.

[2] Stark O (2004). Rethinking the brain drain. World Dev.

[3] Organization, W.H. The world health report 2000: health systems: improving performance. Geneva: World Health Organization; 2000.

[4] Hongoro C, McPake B (2004). How to bridge the gap in human resources for health. Lancet.

[5] Mullan F (2005). The metrics of the physician brain drain. N Engl J Med.

[6] Muula A, Panulo B (2007). Lost investment returns from the migration of medical doctors from Malawi. Tanzan J Health Res.

[7] Tayebi SK, Emadzadeh M, Rostami H (2011). The effect of brain drain on economic growth of developing countries. Quart J Econom Growth Dev Res.

[8] Castro-Palaganas E (2017). An examination of the causes, consequences, and policy responses to the migration of highly trained health personnel from the Philippines: the high cost of living/leaving—a mixed method study. Hum Resour Health.

[9] Pagett C, Padarath A. A review of codes and protocols for the migration of health workers. Harare: EQUINET; 2007. EQUINET Discussion paper No. 50. http://www.equinetafrica.org/bibl/docs/Dis50HRpagett.pdf. Accessed 15 Apr 2008.

[10] Noorbala A (2011). Psychosocial health and strategies for improvement. Iran J Psychiatry Clin Psychol.

[11] Buchan J, Dhillon IS, Campbell J, editors. Health Employment and Economic Growth: An Evidence Base. Geneva: World Health Organization; 2017.

[12] Tehran University of Medical Sciences 2021–2022 Ranking. 2021. Available from: https://cwur.org/2021-22/Tehran-University-of-Medical-Sciences.php.

[13] Asadi H (2017). Factors affecting intent to immigration among Iranian health workers in 2016. Electron Physician.

[14] Lawshe CH (1975). A quantitative approach to content validity. Pers Psychol.

[15] Alaeddini F, Fatemi R, Ranjbaran H, Feiz Zadeh A, Ardalan A, HosseinPoor A, Asghari Roodsari E, Eskandari S, Tavakol HR, Mirzasadeghi AR, Razavi A. The inclination to immigration and the related factors among Iranian physicians. Hakim Res J. 2005;8(3):9–15.

[16] Hossain N, et al. Physicians’ migration: perceptions of Pakistani medical students. J Coll Phys Surg Pak. 2016;26:696–701. 27539766

[17] Sheikh A (2012). Physician migration at its roots: a study on the factors contributing towards a career choice abroad among students at a medical school in Pakistan. Global Health.

[18] Syed NA (2008). Reasons for migration among medical students from Karachi. Med Educ.

[19] Shahraki M, Ghaderi S. Projecting the shortages and surpluses of general practitioners in Iran*.* J Commun Health Res. 2021;10(2):136–49.

[20] Krajewski-Siuda K (2012). Emigration preferences and plans among medical students in Poland. Hum Resour Health.

[21] Polasek O, Kolcic I. Croatia’s brain drain. BMJ. 2005;331(7526):1204–1204. 10.1136/bmj.331.7526.1204 PMC1285110 16293858

[22] Gouda P, et al. Ireland’s medical brain drain: migration intentions of Irish medical students. Hum Resour Health. 2015;13:11–11. 10.1186/s12960-015-0003-9 PMC4363465 25889783

[23] Deressa W, Azazh A (2012). Attitudes of undergraduate medical students of Addis Ababa University towards medical practice and migration. Ethiopia BMC Med Educ.

[24] Eliason S, et al. Migration intentions of Ghanaian medical students: the influence of existing funding mechanisms of medical education (“the fee factor”). Ghana Med J. 2014;48(2):78–84. 10.4314/gmj.v48i2.4 PMC4310336 25667554

[25] Mehdi A. Youth Emigration and the brain drain from iran: reasons, trends and directions. Вестник Нижегородского университета им. НИ Лобачевского. Серия: Социальные науки. 2020(3 (59)):52-9.

[26] Ranjbar F (2015). Fertility behaviour of Iranian women: a community-based cross-sectional study. Arch Iran Med.

[27] Moghadam ZB (2018). Review of the high level of education and reduced fertility in iranian women: have women been empowered. Int J Womens Health Reprod Sci.

[28] Behmanesh F, et al. The relationship of married women’s marriage duration with their reproductive practices. Nursi Midwifery Stud. 2018;7(3):122–7.

[29] Akl EA (2008). Post-graduation migration intentions of students of Lebanese medical schools: a survey study. BMC Public Health.

[30] Sawaf B (2018). Specialty preference and intentions to study abroad of Syrian medical students during the crisis. BMC Med Educ.

[31] Goštautaitė B (2018). Migration intentions of Lithuanian physicians, nurses, residents and medical students. Health Policy.

[32] Umar A. Perception of demographic characteristics of rural-urban migrants in Shani Local Government Area of Borno State, Nigeria. Journal homepage: www.ijrpr.com ISSN. 2582: p. 7421.

[33] Imran N (2012). Brain drain: a harsh reality. International migration of pakistani medical graduates. JPMI.

[34] Santric-Milicevic M (2015). Determinants of intention to work abroad of college and specialist nursing graduates in Serbia. Nurse Educ Today.

[35] Poppe A (2014). Why sub-Saharan African health workers migrate to European countries that do not actively recruit: a qualitative study post-migration. Glob Health Action.

